# Synthesis and crystal structure of NaMgFe(MoO_4_)_3_

**DOI:** 10.1107/S205698901600829X

**Published:** 2016-05-27

**Authors:** Manel Mhiri, Abdessalem Badri, Mongi Ben Amara

**Affiliations:** a Unité de Recherche, Matériaux Inorganiques, Faculté des Sciences, Université de Monastir, 5019 Monastir, Tunisia

**Keywords:** crystal structure, iron molybdate, anionic framework, Na–Fe–Mo–O system

## Abstract

The iron molybdate NaMgFe(MoO_4_)_3_ {sodium magnesium iron(III) tris­[molybdate(VI)]} has been synthesized by the flux method. This compound is isostructural with α-NaFe_2_(MoO_4_)_3_ and crystallizes in the triclinic space group *P*-1. Its structure is built up from [Mg,Fe]_2_O_10_ units of edge-sharing [Mg,Fe]O_6_ octa­hedra which are linked to each other through the common corners of [MoO_4_] tetra­hedra. The resulting anionic three-dimensional framework leads to the formation of channels along the [101] direction in which the Na^+^ cations are located.

## Abstract 2

The iron molybdate NaMgFe(MoO_4_)_3_ is isostructural with α-NaFe_2_(MoO_4_)_3_ and its structure is built up from [Mg,Fe]_2_O_10_ units of edge-sharing [Mg,Fe]O_6_ octa­hedra which are linked to each other through the common corners of [MoO_4_] tetra­hedra. The resulting anionic three-dimensional framework leads to the formation of channels along the [101] direction, where the Na^+^ cations are located.

## Chemical context   

Iron molybdates have been subject to very intensive research as a result of their numerous applications including as catalysts (Tian *et al.*, 2011 ▸), multiferroic properties and more recently as a possible positive electrode in rechargeable batteries (Sinyakov *et al.*, 1978 ▸; Mączka *et al.*, 2011 ▸; Devi & Varadaraju, 2012 ▸). In these materials, the anionic framework is constructed from MoO_4_ tetra­hedra linked to the iron coordination polyhedra, leading to a large variety of crystal structures with a high capacity for cationic and anionic substitutions.

Until now, a total of six orthomolybdate compounds have been reported in the Na–Fe–Mo–O system: Na_9_Fe(MoO_4_)_6_ (Savina *et al.*, 2013 ▸); NaFe(MoO_4_)_2_ (Klevtsova, 1975 ▸); α-NaFe_2_(MoO_4_)_3_, β-NaFe_2_(MoO_4_)_3_ and Na_3_Fe_2_(MoO_4_)_3_ (Muessig *et al.*, 2003 ▸); NaFe_4_(MoO_4_)_5_ (Ehrenberg *et al.*, 2006 ▸). Their structures are described in terms of three-dimensional networks of isolated [MoO_4_] tetra­hedra and [FeO_6_] octa­hedra. The sodium and mixed-valence iron molybdate NaFe_2_(MoO_4_)_3_ exhibits two polymorphs, both crystallizing in the triclinic system. The low-temperature α-phase changes irreversibly at high temperature into a β-phase. In addition to these orthomolybdate compounds, another phase with the formula Na_3_Fe_2_Mo_5_O_16_ and with layers of Mo_3_O_13_ units consisting of [MoO_6_] octa­hedra has been synthesized and characterized (Bramnik *et al.*, 2003 ▸). In addition, Kozhevnikova & Imekhenova (2009 ▸) have investigated the Na_2_MoO_4_–*M*MoO_4_–Fe_2_(MoO_4_)_3_ system (*M* = Mg, Mn, Ni, Co) and have attributed the Nasicon-type structure with space group *R* 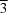 *c* (Kotova & Kozhevnikova, 2003 ▸; Kozhevnikova & Imekhenova, 2009 ▸) to the phase of variable composition Na_(1−*x*)_ *M*_(1−*x*)_Fe_(1+*x*)_(MoO_4_)_3_. More recently, NaNiFe(MoO_4_)_3_ and NaZnFe(MoO_4_)_3_ (Mhiri *et al.*, 2015 ▸) were found to be isostructural to β-NaFe_2_(MoO_4_)_3_ and to have a good ionic conductivity with low activation energy, close to those of Nasicon-type compounds with similar formula such as *A*Zr_2_(PO_4_)_3_ (*A* = Na, Li). As an extension of the previous work, we report here on the synthesis and characterization by X-ray diffraction of a new compound, NaMgFe(MoO_4_)_3_, which is isostructural with α-NaFe_2_(MoO_4_)_3_.

## Structural commentary   

**Figure 1:**
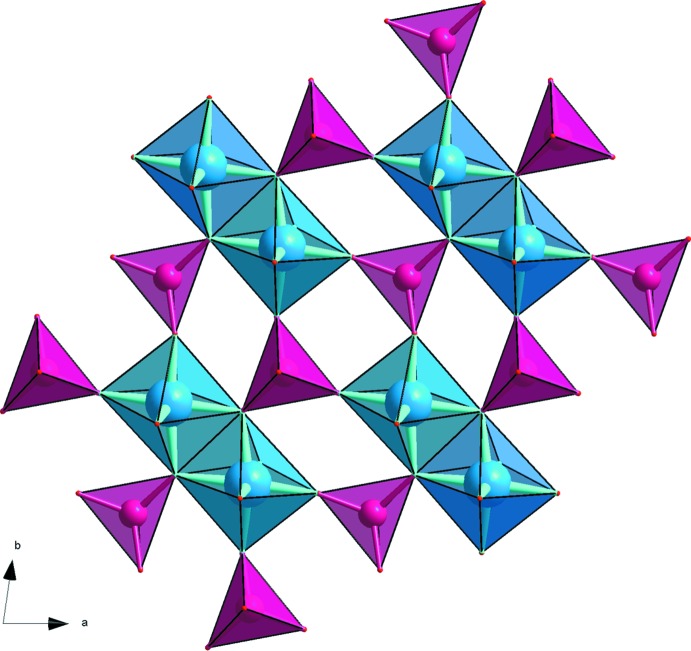
[Mg,Fe]_2_O_10_ units parallel to [1
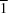
0] in NaMgFe(MoO_4_)_3_ structure. [Mg,Fe]_2_O_10_ dimers are shown in blue and MoO_4_ tetra­hedra in purple.

**Figure 2:**
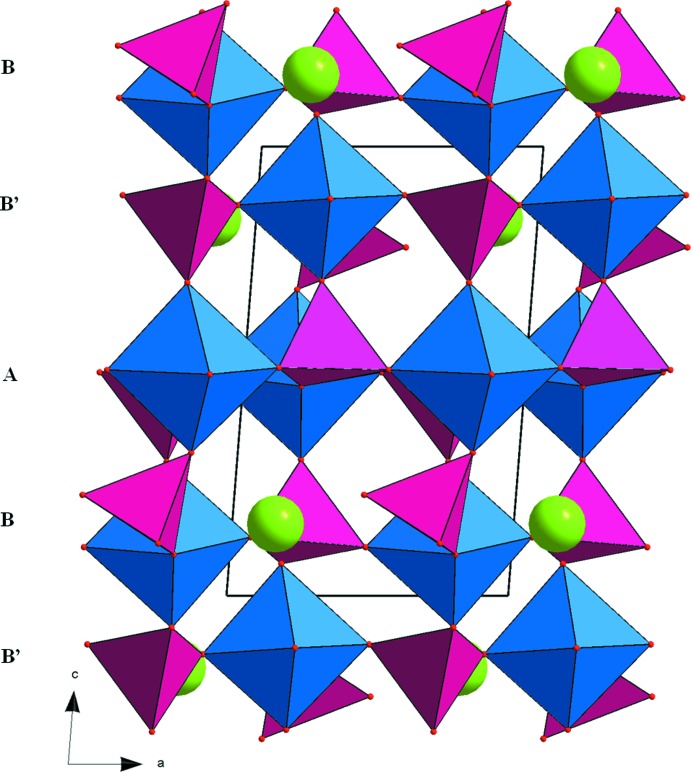
Projection of the NaMgFe(MoO_4_)_3_ structure along the *b* axis. [Mg,Fe]_2_O_10_ dimers are shown in blue; MoO_4_ tetra­hedra in purple and Na^+^ cations as green spheres.

**Figure 3:**
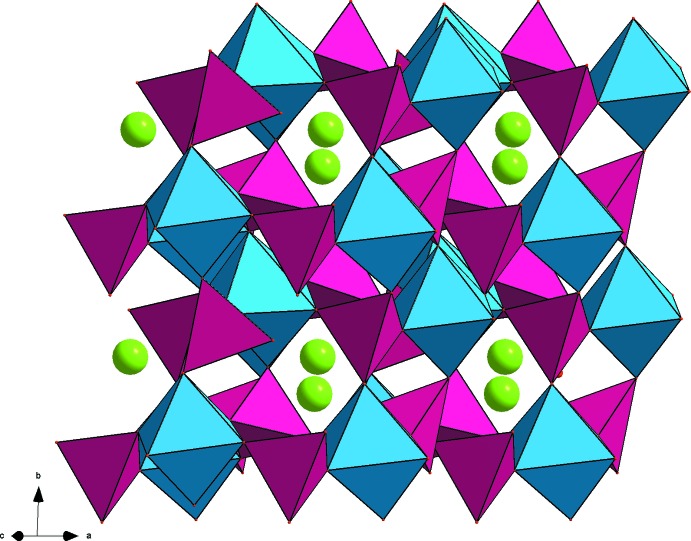
Channels along [101] in the structure of NaMgFe(MoO_4_)_3_. [Mg,Fe]_2_O_10_ dimers are shown in blue, MoO_4_ tetra­hedra in purple and Na^+^ cations as green spheres.

The title NaMgFe(MoO_4_)_3_ structure is based on a three-dimensional framework of [Mg,Fe]_2_O_10_ units of edge-sharing [Mg,Fe]O_6_ octa­hedra, connected to each other through the common corners of [MoO_4_] tetra­hedra. All [Mg,Fe]_2_O_10_ units are parallel to [1
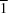
0] (Fig. 1 ▸). In this structure, two types of layers (*A* and *B*), similar to those observed in α-NaFe_2_(MoO_4_)_3_, are aligned parallel to (110) with the sequence –*A*–*B*–*B*′–*A*–*B*–*B*′– and stacked along [001]. *B*′ layers are obtained from *B* by an inversion centre located on the *A* planes (Fig. 2 ▸). The resulting anionic three-dimensional framework leads to the formation of channels along [101] in which the sodium ions are located (Fig. 3 ▸).

**Figure 4:**
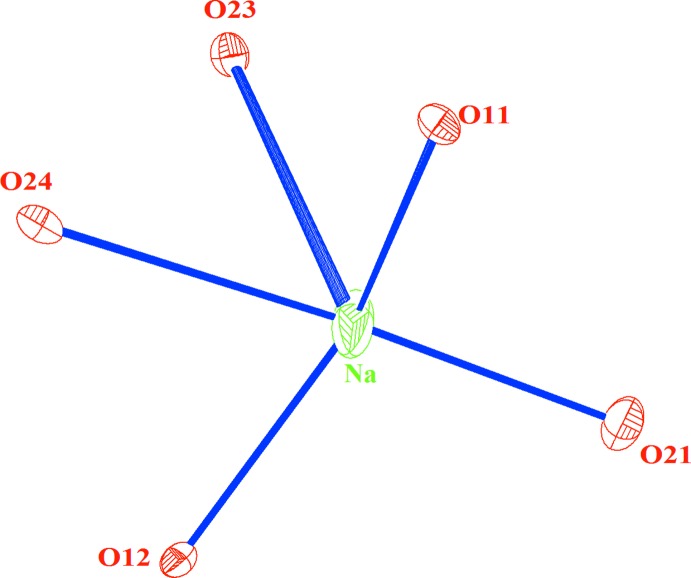
The environment of the Na^+^ cation showing displacement ellipsoids drawn at the 50% probability level.

In the title structure, all atoms are located in general positions. The three crystallographically different molybdenum atoms have a tetra­hedral coordination with Mo—O distances between 1.715 (3) and 1.801 (2) Å. The mean distances (Mo1—O = 1.762, Mo2—O = 1.766 and Mo3—O = 1.760 Å) are in good accordance with those usually observed in molybdates (Abrahams *et al.*, 1967 ▸; Harrison & Cheetham, 1989 ▸; Smit *et al.*, 2006 ▸). The [Mg,Fe]—O distances and the *cis* O—[Mg,Fe]—O angles in the [Mg,Fe]_2_O_10_ units range from 2.003 (3) to 2.099 (3) Å and from 81.2 (1) to 177.8 (1)°, respectively. This dispersion reflects a slight distortion of the [Mg,Fe]O_6_ octa­hedra. The average distances [Mg,Fe]1—O = 2.059 and [Mg,Fe]2—O = 2.013 Å lie between the values of 1.990 Å observed for six-coordinated Fe^3+^ in LiFe(MoO_4_)_2_ (van der Lee *et al.* 2008 ▸) and 2.072 Å reported for Mg^2+^ with the same coordination in NaMg_3_Al(MoO_4_)_5_ (Hermanowicz *et al.*, 2006 ▸). This result is related to the disordered distribution of Fe^3+^ and Mg^2+^ in both sites. Assuming sodium–oxygen distances below 3.13 Å (Donnay & Allmann, 1970 ▸), the Na site is surrounded by five oxygen atoms (Fig. 4 ▸).

## Synthesis and crystallization   

Crystals of the title compound were grown in a flux of sodium dimolybdate Na_2_Mo_2_O_7_ with an atomic ratio Na:Mg:Fe:Mo = 5:1:1:7. Appropriate amounts of the starting reactants NaNO_3_, Mg(NO_3_)_2_·6H_2_O, Fe(NO_3_)_3_·9H_2_O and (NH_4_)_6_Mo_7_O_24_·4H_2_O were dissolved in nitric acid and the resulting solution was evaporated to dryness. The dry residue was then placed in a platinum crucible and slowly heated in air up to 673 K for 24 h to remove H_2_O and NH_3_. The mixture was ground in an agate mortar, melted for 2 h at 1123 K and then cooled to room temperature at a rate of 5 K h^−1^. Crystals without regular shape were separated from the flux by washing in boiling water.

## Refinement   

Crystal data, data collection and structure refinement details are summarized in Table 1 ▸. The application of the direct methods revealed two sites, labeled *M*(1) and *M*(2), statistic­ally occupied by the Fe^3+^ and Mg^2+^ ions. This distribution was supported by the *M*1—O and *M*2—O distances which are between the classical values for pure Mg—O and Fe—O bonds. Succeeding difference Fourier synthesis led to the positions of all the remaining atoms.

## Supplementary Material

Crystal structure: contains datablock(s) global, I. DOI: 10.1107/S205698901600829X/br2259sup1.cif

Structure factors: contains datablock(s) I. DOI: 10.1107/S205698901600829X/br2259Isup2.hkl

CCDC reference: 1481125

Additional supporting information:  crystallographic information; 3D view; checkCIF report

## Figures and Tables

**Table 1:** Experimental details

Crystal data	
Chemical formula	NaMgFe(MoO_4_)_3_
*M*_r_	582.97
Crystal system, space group	Triclinic, *P* 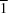
Temperature (K)	293
*a*, *b*, *c* (Å)	6.900 (4), 6.928 (1), 11.055 (1)
α, β, γ (°)	80.24 (1), 83.55 (2), 80.22 (3)
*V* (Å^3^)	511.3 (3)
*Z*	2
Radiation type	Mo *K*α
μ (mm^−1^)	5.15
Crystal size (mm)	0.28 × 0.14 × 0.07

Data collection	
Diffractometer	Enraf–Nonius TurboCAD-4
Absorption correction	ψ scan (North *et al.*, 1968 ▸)
*T*_min_, *T*_max_	0.478, 0.695
No. of measured, independent and observed [*I* > 2σ(*I*)] reflections	3429, 2983, 2850
*R*_int_	0.014
(sin θ/λ)_max_ (Å^−1^)	0.703

Refinement	
*R*[*F* ^2^ > 2σ(*F* ^2^)], *wR*(*F* ^2^), *S*	0.025, 0.068, 1.19
No. of reflections	2983
No. of parameters	168
No. of restraints	4
Δρ_max_, Δρ_min_ (e Å^−3^)	1.47, −1.60

Computer programs: *CAD-4 EXPRESS* (Enraf–Nonius, 1994 ▸), *XCAD4* (Harms & Wocadlo, 1995 ▸), *SIR92* (Altomare *et al.*, 1993 ▸), *SHELXL2014*/7 (Sheldrick, 201 ▸), *DIAMOND* (Brandenburg & Putz, 1999 ▸) and *WinGX* publication routines (Farrugia, 2012 ▸).

## supplementary crystallographic information

### Crystal data

**Table:**

FeMgMo_3_NaO_12_	*Z* = 2
*M**_r_* = 582.97	*F*(000) = 542
Triclinic, *P*1	*D*_x_ = 3.786 Mg m^−^^3^
*a* = 6.900 (4) Å	Mo *K*α radiation, λ = 0.71073 Å
*b* = 6.928 (1) Å	Cell parameters from 25 reflections
*c* = 11.055 (1) Å	θ = 9.1–11.4°
α = 80.24 (1)°	µ = 5.15 mm^−^^1^
β = 83.55 (2)°	*T* = 293 K
γ = 80.22 (3)°	Prism, brown
*V* = 511.3 (3) Å^3^	0.28 × 0.14 × 0.07 mm

### Data collection

**Table:**

Enraf–Nonius TurboCAD-4 diffractometer	*R*_int_ = 0.014
Radiation source: fine-focus sealed tube	θ_max_ = 30.0°, θ_min_ = 3.0°
non–profiled ω/2τ scans	*h* = −9→9
Absorption correction: ψ scan (North *et al.*, 1968)	*k* = −9→9
*T*_min_ = 0.478, *T*_max_ = 0.695	*l* = −1→15
3429 measured reflections	2 standard reflections every 120 min
2983 independent reflections	intensity decay: 1.1%
2850 reflections with *I* > 2σ(*I*)	

### Refinement

**Table:**

Refinement on *F*^2^	Primary atom site location: structure-invariant direct methods
Least-squares matrix: full	Secondary atom site location: difference Fourier map
*R*[*F*^2^ > 2σ(*F*^2^)] = 0.025	*w* = 1/[σ^2^(*F*_o_^2^) + (0.0308*P*)^2^ + 2.3858*P*] where *P* = (*F*_o_^2^ + 2*F*_c_^2^)/3
*wR*(*F*^2^) = 0.068	(Δ/σ)_max_ = 0.001
*S* = 1.19	Δρ_max_ = 1.47 e Å^−^^3^
2983 reflections	Δρ_min_ = −1.60 e Å^−^^3^
168 parameters	Extinction correction: *SHELXL2014*/7 (Sheldrick 2015), Fc^*^=kFc[1+0.001xFc^2^λ^3^/sin(2θ)]^-1/4^
4 restraints	Extinction coefficient: 0.0074 (5)

### Special details

**Table:**

Geometry. All esds (except the esd in the dihedral angle between two l.s. planes) are estimated using the full covariance matrix. The cell esds are taken into account individually in the estimation of esds in distances, angles and torsion angles; correlations between esds in cell parameters are only used when they are defined by crystal symmetry. An approximate (isotropic) treatment of cell esds is used for estimating esds involving l.s. planes.
Refinement. Refinement of F^2^ against ALL reflections. The weighted R-factor wR and goodness of fit S are based on F^2^, conventional R-factors R are based on F, with F set to zero for negative F^2^. The threshold expression of F^2^ > 2sigma(F^2^) is used only for calculating R-factors(gt) etc. and is not relevant to the choice of reflections for refinement. R-factors based on F^2^ are statistically about twice as large as those based on F, and R- factors based on ALL data will be even larger.

### Fractional atomic coordinates and isotropic or equivalent isotropic displacement parameters (Å^2^)

**Table:**

	*x*	*y*	*z*	*U*_iso_*/*U*_eq_	Occ. (<1)
Na	0.8586 (4)	0.5914 (4)	0.8148 (4)	0.0757 (12)	
Mg1	0.8152 (1)	0.1703 (1)	0.50854 (8)	0.00851 (16)	0.7558 (7)
Fe1	0.8152 (1)	0.1703 (1)	0.50854 (8)	0.00851 (16)	0.2442 (7)
Mg2	0.77528 (8)	0.77491 (8)	0.11025 (5)	0.00785 (12)	0.2442 (7)
Fe2	0.77528 (8)	0.77491 (8)	0.11025 (5)	0.00785 (12)	0.7558 (7)
Mo1	0.75910 (4)	0.10066 (4)	0.85110 (2)	0.00799 (8)	
O11	0.8166 (4)	0.8508 (4)	0.9264 (2)	0.0125 (5)	
O12	0.9297 (4)	0.2547 (4)	0.8737 (3)	0.0146 (5)	
O13	0.5170 (4)	0.2053 (4)	0.8938 (3)	0.0158 (5)	
O14	0.7784 (5)	0.0889 (4)	0.6953 (2)	0.0185 (5)	
Mo2	0.70522 (4)	0.28318 (4)	0.18835 (3)	0.00950 (8)	
O21	0.4579 (4)	0.3458 (5)	0.2289 (3)	0.0232 (6)	
O22	0.7436 (4)	0.0675 (4)	0.1148 (3)	0.0185 (5)	
O23	0.8372 (4)	0.2322 (4)	0.3205 (2)	0.0173 (5)	
O24	0.8015 (4)	0.4878 (4)	0.0918 (2)	0.0148 (5)	
Mo3	0.27372 (4)	0.29658 (4)	0.54507 (2)	0.00732 (8)	
O31	0.1224 (4)	0.1328 (4)	0.5056 (2)	0.0113 (4)	
O32	0.2458 (5)	0.2976 (4)	0.7045 (2)	0.0194 (5)	
O33	0.5183 (4)	0.2083 (4)	0.5042 (3)	0.0172 (5)	
O34	0.2067 (4)	0.5383 (4)	0.4690 (3)	0.0153 (5)	

### Atomic displacement parameters (Å^2^)

**Table:**

	*U*^11^	*U*^22^	*U*^33^	*U*^12^	*U*^13^	*U*^23^
Na	0.0293 (12)	0.0255 (11)	0.186 (4)	0.0089 (9)	−0.0496 (18)	−0.0429 (17)
Mg1	0.0079 (4)	0.0081 (4)	0.0091 (4)	−0.0007 (3)	−0.0018 (3)	0.0001 (3)
Fe1	0.0079 (4)	0.0081 (4)	0.0091 (4)	−0.0007 (3)	−0.0018 (3)	0.0001 (3)
Mg2	0.0080 (2)	0.0073 (2)	0.0079 (2)	−0.00174 (18)	−0.00177 (18)	0.00103 (18)
Fe2	0.0080 (2)	0.0073 (2)	0.0079 (2)	−0.00174 (18)	−0.00177 (18)	0.00103 (18)
Mo1	0.00697 (13)	0.00870 (13)	0.00770 (13)	−0.00175 (9)	−0.00095 (9)	0.00122 (9)
O11	0.0163 (12)	0.0106 (11)	0.0094 (11)	−0.0013 (9)	−0.0021 (9)	0.0019 (8)
O12	0.0112 (11)	0.0152 (12)	0.0182 (12)	−0.0052 (9)	−0.0040 (9)	0.0003 (10)
O13	0.0090 (11)	0.0178 (12)	0.0194 (13)	−0.0009 (9)	−0.0016 (9)	−0.0005 (10)
O14	0.0258 (14)	0.0185 (13)	0.0092 (11)	−0.0021 (11)	−0.0017 (10)	0.0018 (10)
Mo2	0.01041 (14)	0.00802 (13)	0.00985 (13)	−0.00235 (9)	−0.00173 (9)	0.00076 (9)
O21	0.0133 (12)	0.0295 (16)	0.0261 (15)	−0.0028 (11)	−0.0017 (11)	−0.0026 (12)
O22	0.0226 (14)	0.0110 (12)	0.0229 (14)	−0.0050 (10)	−0.0032 (11)	−0.0017 (10)
O23	0.0192 (13)	0.0188 (13)	0.0125 (12)	−0.0009 (10)	−0.0033 (10)	0.0005 (10)
O24	0.0203 (13)	0.0100 (11)	0.0129 (11)	−0.0016 (9)	0.0019 (9)	−0.0012 (9)
Mo3	0.00771 (13)	0.00787 (13)	0.00674 (13)	−0.00290 (9)	−0.00105 (9)	−0.00015 (9)
O31	0.0093 (10)	0.0089 (10)	0.0167 (12)	−0.0027 (8)	−0.0026 (9)	−0.0027 (9)
O32	0.0266 (15)	0.0239 (14)	0.0088 (11)	−0.0081 (11)	−0.0019 (10)	−0.0010 (10)
O33	0.0107 (11)	0.0198 (13)	0.0212 (13)	−0.0025 (10)	−0.0018 (10)	−0.0025 (10)
O34	0.0189 (13)	0.0086 (11)	0.0179 (12)	−0.0027 (9)	−0.0027 (10)	0.0008 (9)

### Geometric parameters (Å, º)

**Table:**

Na—O21^i^	2.244 (4)	Mg2—O32^i^	2.019 (3)
Na—O12	2.296 (4)	Mg2—O12^ii^	2.036 (3)
Na—O11	2.308 (4)	Mo1—O14	1.727 (3)
Na—O24^ii^	2.604 (4)	Mo1—O13	1.751 (3)
Na—O23^ii^	2.772 (5)	Mo1—O12	1.780 (3)
Mg1—O33	2.025 (3)	Mo1—O11^vii^	1.789 (3)
Mg1—O23	2.044 (3)	Mo2—O21	1.715 (3)
Mg1—O14	2.045 (3)	Mo2—O23	1.761 (3)
Mg1—O34^i^	2.054 (3)	Mo2—O22	1.787 (3)
Mg1—O31^iii^	2.089 (3)	Mo2—O24	1.799 (3)
Mg1—O31^iv^	2.099 (3)	Mo3—O33	1.731 (3)
Mg2—O13^i^	2.003 (3)	Mo3—O32	1.753 (3)
Mg2—O24	2.009 (3)	Mo3—O34	1.753 (3)
Mg2—O22^v^	2.010 (3)	Mo3—O31	1.801 (2)
Mg2—O11^vi^	2.012 (3)		
			
O21^i^—Na—O12	106.29 (15)	O24—Mg2—O11^vi^	90.58 (11)
O21^i^—Na—O11	92.34 (14)	O22^v^—Mg2—O11^vi^	85.22 (11)
O12—Na—O11	131.5 (2)	O13^i^—Mg2—O32^i^	91.56 (12)
O21^i^—Na—O24^ii^	169.3 (2)	O24—Mg2—O32^i^	90.53 (12)
O12—Na—O24^ii^	71.63 (12)	O22^v^—Mg2—O32^i^	93.77 (12)
O11—Na—O24^ii^	81.96 (12)	O11^vi^—Mg2—O32^i^	175.79 (12)
O21^i^—Na—O23^ii^	125.19 (19)	O13^i^—Mg2—O12^ii^	176.14 (11)
O12—Na—O23^ii^	115.39 (14)	O24—Mg2—O12^ii^	90.70 (12)
O11—Na—O23^ii^	85.84 (12)	O22^v^—Mg2—O12^ii^	91.08 (12)
O24^ii^—Na—O23^ii^	63.66 (10)	O11^vi^—Mg2—O12^ii^	91.24 (11)
O33—Mg1—O23	88.07 (12)	O32^i^—Mg2—O12^ii^	84.69 (12)
O33—Mg1—O14	88.89 (12)	O14—Mo1—O13	108.16 (14)
O23—Mg1—O14	174.80 (12)	O14—Mo1—O12	106.87 (14)
O33—Mg1—O34^i^	89.20 (12)	O13—Mo1—O12	110.69 (13)
O23—Mg1—O34^i^	93.92 (12)	O14—Mo1—O11^vii^	106.05 (13)
O14—Mg1—O34^i^	90.26 (12)	O13—Mo1—O11^vii^	111.66 (13)
O33—Mg1—O31^iii^	177.80 (12)	O12—Mo1—O11^vii^	113.08 (12)
O23—Mg1—O31^iii^	89.73 (11)	O21—Mo2—O23	110.07 (14)
O14—Mg1—O31^iii^	93.30 (12)	O21—Mo2—O22	109.36 (15)
O34^i^—Mg1—O31^iii^	91.09 (11)	O23—Mo2—O22	109.03 (13)
O33—Mg1—O31^iv^	98.59 (12)	O21—Mo2—O24	110.70 (14)
O23—Mg1—O31^iv^	88.75 (11)	O23—Mo2—O24	105.75 (13)
O14—Mg1—O31^iv^	87.53 (11)	O22—Mo2—O24	111.86 (13)
O34^i^—Mg1—O31^iv^	171.86 (11)	O33—Mo3—O32	108.10 (14)
O31^iii^—Mg1—O31^iv^	81.22 (11)	O33—Mo3—O34	110.71 (13)
O13^i^—Mg2—O24	88.40 (12)	O32—Mo3—O34	109.21 (14)
O13^i^—Mg2—O22^v^	90.10 (12)	O33—Mo3—O31	108.43 (13)
O24—Mg2—O22^v^	175.47 (12)	O32—Mo3—O31	109.96 (13)
O13^i^—Mg2—O11^vi^	92.52 (11)	O34—Mo3—O31	110.40 (12)

Symmetry codes: (i) −*x*+1, −*y*+1, −*z*+1; (ii) −*x*+2, −*y*+1, −*z*+1; (iii) *x*+1, *y*, *z*; (iv) −*x*+1, −*y*, −*z*+1; (v) *x*, *y*+1, *z*; (vi) *x*, *y*, *z*−1; (vii) *x*, *y*−1, *z*.
